# Spinal tuberculosis, pathophysiology and radiological presentation, three case reports

**DOI:** 10.7705/biomedica.7685

**Published:** 2025-09-22

**Authors:** Vanessa M. S. Ross, Bibiana Pinzón, Diana María Palacios-Ortiz, Zandra De La Rosa-Noriega, Jana Abi Rafeh, Leonardo F. Jurado

**Affiliations:** 1 Faculty of Medicine and Health Sciences, McGill University, Montreal, Canada McGill University Faculty of Medicine and Health Sciences McGill University Montreal Canada; 2 Departamento de Radiología, Hospital Universitario Fundación Santa Fe de Bogotá, Bogotá, D. C., Colombia Departamento de Radiología Hospital Universitario Fundación Santa Fe de Bogotá Bogotá D. C Colombia; 3 Departamento de Patología y Laboratorios Hospital Universitario, Fundación Santa Fe de Bogotá, Bogotá, D.C., Colombia Departamento de Patología y Laboratorios Hospital Universitario Fundación Santa Fe de Bogotá Bogotá D.C Colombia; 4 Department of Experimental Medicine, McGill University Health Centre, Meakins-Christie Laboratories, McGill University, Montreal, Canada McGill University Department of Experimental Medicine McGill University Montreal Canada; 5 Department of Pathology, McGill University Health Centre, McGill International TB Centre, Meakins-Christie Laboratories, McGill University, Montreal, Canada McGill University McGill University Montreal Canada

**Keywords:** Mycobacterium tuberculosis, spinal tuberculosis, Pott's disease, physiopathology, Mycobacterium tuberculosis, tuberculosis vertebral, mal de Pott, fisiopatología

## Abstract

Prompt diagnosis and treatment of spinal tuberculosis are key in preventing its neurological and physical sequelae. This affection, also known as Pott's disease, should be considered a differential diagnosis in patients presenting with unexplained back pain that can lead to neurological symptoms and eventually paraplegia.

*Mycobacterium tuberculosis,* the etiological agent of tuberculosis, spreads from the lungs to the spine via venous or arterial pathways, causing lesions apparent upon imaging. Radiological findings include osseous destruction, disk collapse, abscess formation, and spinal deformity. While magnetic resonance is considered the most sensitive and specific imaging modality to establish a diagnosis, plain radiographs and computed tomography can provide useful information.

This manuscript discusses three Colombian cases of spinal tuberculosis with the goal of increasing familiarity regarding the pathophysiology, clinical and radiological manifestations, and differential diagnosis of this rare but potentially devastating disease.

## Resumen

El diagnóstico y el manejo oportuno de la tuberculosis vertebral son fundamentales en la prevención de sus secuelas físicas y neurológicas. Esta enfermedad, también conocida como el mal de Pott, debe ser considerada como diagnóstico diferencial en los pacientes con dolor de espalda sin causa evidente, eventuales síntomas neurológicos y paraplejía. *Mycobacterium tuberculosis,* el agente etiológico de la tuberculosis, se disemina desde los pulmones a la columna vertebral por vía arterial o venosa, causando lesiones evidentes radiológicamente. Los hallazgos imagenológicos incluyen destrucción ósea, colapso discal, formación de abscesos y deformidad espinal. Aunque la resonancia magnética se considera la modalidad más adecuada para lograr el diagnostico, la radiografía simple y la tomografia computarizada pueden suministrar información útil.

Se discuten tres casos de pacientes colombianos con tuberculosis vertebral con el objetivo de que el lector se familiarice con la fisiopatología, la presentación clínica y la radiológica, así como el diagnóstico de esta enfermedad infrecuente pero potencialmente devastadora.

Tuberculosis is considered the deadliest transmissible disease worldwide, with an estimated incidence of over 10 million people in 2021 ^1^. It is caused by *Mycobacterium tuberculosis* and is transmitted through airborne particles ^2^. In most cases (~80%), the lungs are affected, causing coughing, expectorations, weight loss, chest pain, fever, and impairment of general condition ^2^. However, *M. tuberculosis* can disseminate through the blood to various organs ^3^. In some cases, this dissemination causes no evident respiratory involvement ^4^.

Extra-pulmonary tuberculosis accounts for ~20% of tuberculosis cases ^3^. It can develop as a progression of a primary infection or reactivation of a latent infection ^3^. During the primary infection, in which the immune system is first exposed to the pathogen, patients may experience flu-like symptoms such as fever, cough, and fatigue. In most immunocompetent individuals, this phase leads to asymptomatic latent infection. When the immune system cannot control the infection, active tuberculosis occurs with the clinical presentation described. This can occur right after primary infection or after years of latent infection when the immune system weakens.

Skeletal tuberculosis represents ~10-35% of extra-pulmonary tuberculosis cases and 2% of tuberculosis cases in the United States ^5^. It most commonly affects the lower thoracic and upper lumbar regions of the spine, as well as the hip joints ^3^^-^^5^. Spinal tuberculosis, accounts for 50% of all cases of bone and joint tuberculosis ^4^. This infection, also known as Pott's disease from Sir Percival Pott's description of the illness in 1779, most commonly presents itself as worsening back pain ^4^^,^^5^. Patients may experience vertebral collapse, which causes spinal deformity and damages to the spinal cord, eventually resulting in paraplegia ^4^^,^^5^.

In this article, we describe three Colombian cases of spinal tuberculosis. Through discussion focused on clinical, physio-pathological and radiological characteristics, our purpose is to help increase awareness of the presentation of Pott's disease to facilitate early diagnosis. Rapid identification of the illness and prompt treatment are important to avoid permanent neurological sequelae and spinal deformity.

## Case one

A 66-year-old female presented to the emergency department with a two-week history of lumbar back-pain, limited mobility, and no relief from analgesics. She had been hospitalized recently for spondylodiscitis at T_10_-T_11_, due to rifampin-resistant tuberculosis and paravertebral abscess, which was confirmed by biopsy and GeneXpert analysis, with no radiological evidence of pulmonary involvement. The current pain was accompanied by bilateral lower-limb paresthesia.

**Figure 1:**
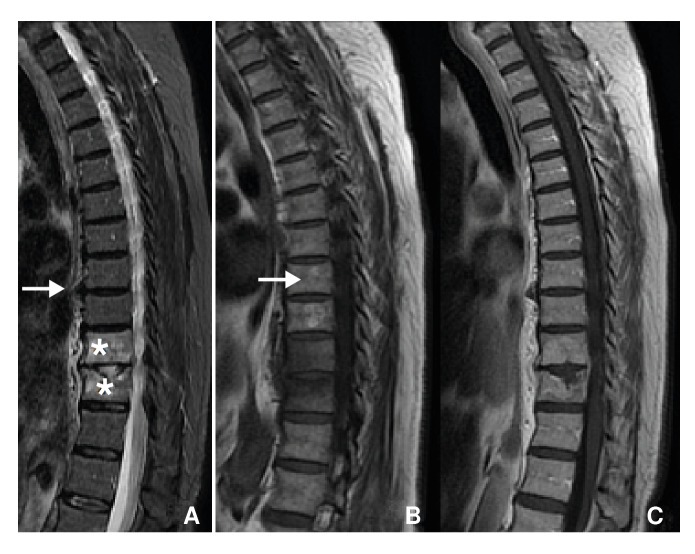
Patient one. Magnetic resonance of thoracic spine. A) Sagital T_2_-weighted image with fat saturation showing edema of vertebrae T_10_, T_11_, and intervertebral disc (asterisk). Sagital T_1_-weighted images with (B) and without (C) contrast evidencing endplate and disc enhancement due to spondylodiscitis, without abscesses (white arrows).

Upon physical examination, pain was elicited by palpation of the lumbar spine, with left predominance. No deficits were found in muscle strength testing nor neurovascular status of lower limbs. Initial bloodwork did not indicate any leukocytosis or inflammatory response. Initial imaging studies, including X-rays and magnetic resonance imaging (MRI), revealed recurrence of tuberculous spondylodiscitis at T_10_-T_11_ resulting in fracture and spinal cord compression (figure 1).

The patient underwent a surgical debridement with posterior corpectomy T_10_-T_11_, decompression laminectomy and transpedicular arthrodesis T_8_-T_9_ to T_12_-L_1_, intracorporal arthrodesis, and bone graft. The patient began treatment with a five-drug anti-tuberculous therapy, including clofazimine, levofloxacin, linezolid, and bedaquiline, for 9 months, leading to curation of the disease.

## Case two

**Figure 2:**
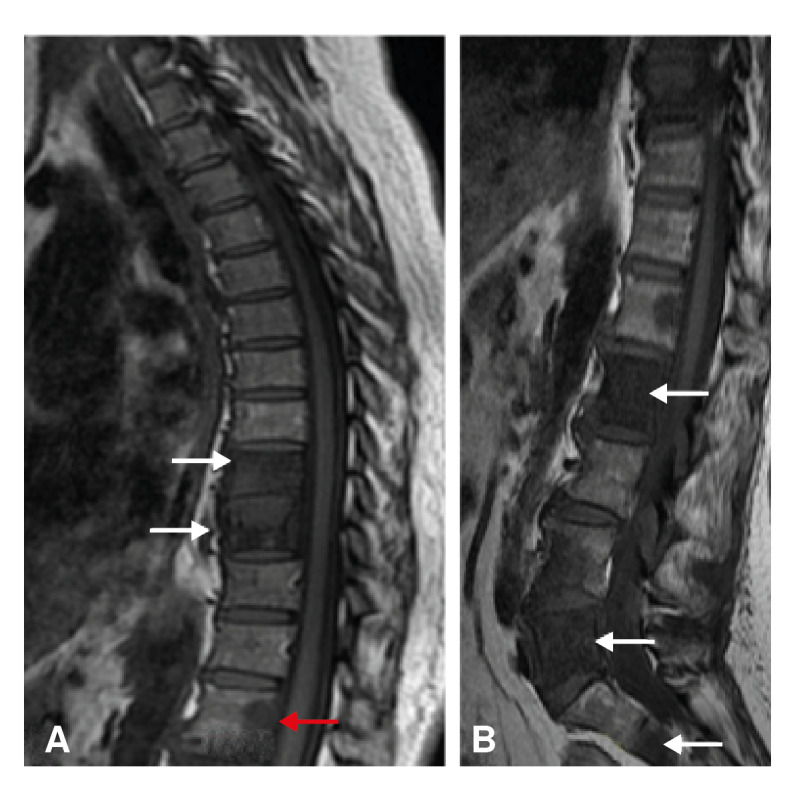
Patient two. Magnetic resonance. Sagital T_1_-weighted image of thoracic (A) and lumbar spine (B) showing decreased signal intensity of vertebral bodies T_9_, T_10_, L_4_, L_5_, and S_2_, with a focal alteration in L_1_ (red arrow). Findings show bone marrow edema (white arrows).

A 61-year-old female presented to the emergency department due to a lumbar pain exacerbation, now radiating to the abdominal region. The lumbar pain had been present for a year. Previously, osteogenic lesions in thoracic and lumbar vertebrae (figure 2), as well as the iliac bone, sacrum, and right humerus, had been described. Metastases from an undiscovered primary tumor was the primary suspicion for these findings. The patient was given analgesics until a diagnosis could be established.

**Figure 3:**
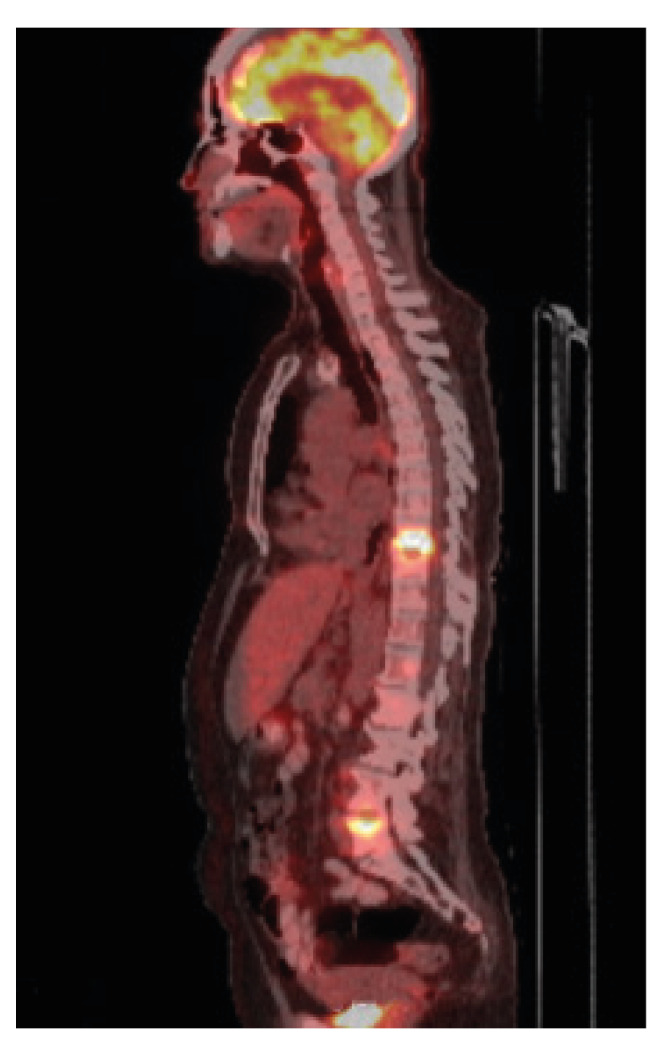
Patient two. Fluoro-deoxy-glucose positron emission tomography (FDG-PET). Increased FDG uptake was observed at T_9_, T_10_, and L_5_ with a maximum standardized uptake value of 20. This finding represents an inflammatory process in which cells consume high levels of glucose.

Pulmonary X-ray was normal. A fluoro-deoxy-glucose (FDG) positron emission tomography computerized tomography (PET-CT) showed increased uptake in T_9_-T_10_, L_2_-L_3_ and L_5_, as well as in the posterior region of the right iliac bone and the right humeral head (figure 3).

Physical examination revealed inguinal lymphadenopathies. A biopsy was performed with the objective of establishing a diagnosis. Histopathology of the lymph nodes showed a chronic granulomatous disease with acid-fast bacilli, confirming the diagnosis of Pott's disease.

**Figure 4:**
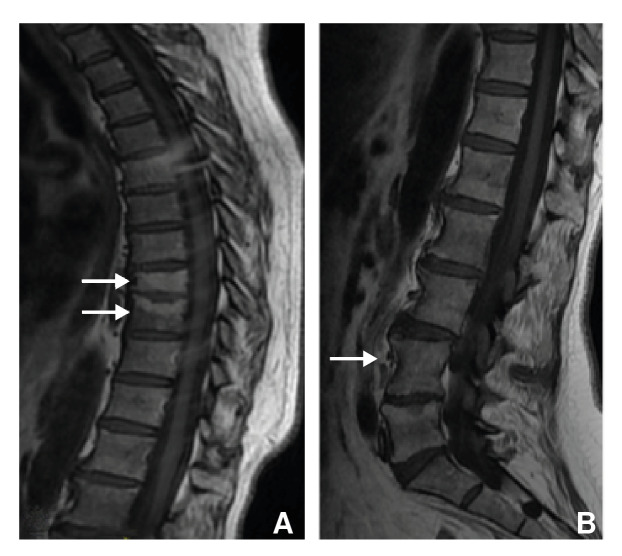
Magnetic resonance. Control after treatment. Sagital T_1_-weighted image of the thoracic (A) and lumbar (B) spine. Areas with blastic appearance and increased signal intensity on T_9_, T_10_, and the vertebral endplate of L_5_ represent bone marrow fat replacement without edema.

The patient received antituberculosis treatment for six months, resulting in full recovery (figure 4).

## Case three

A 37-year-old female presented to the emergency department with a 5-month history of lower lumbar pain. She had consulted several times, only receiving pain treatment, with no clinical improvement.

**Figure 5:**
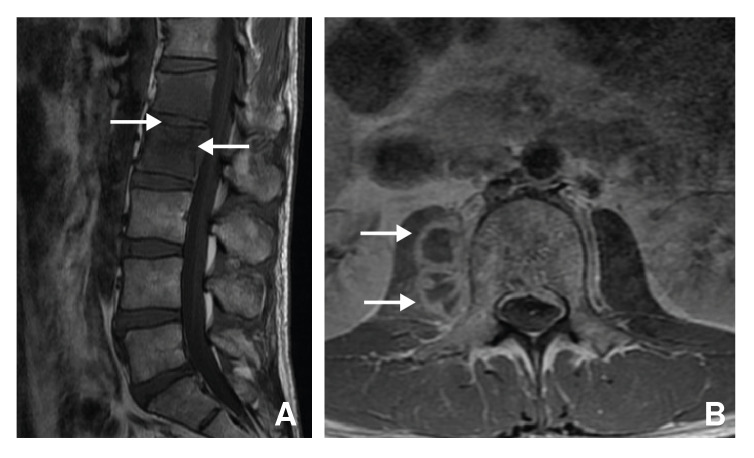
Patient three. Magnetic resonance of the lumbar spine with gadolinium. A. Spondylodiscitis in L_1_ and L_2_ with epidural abscess (arrows); B) Axial plane in L_2_ showing an extension of the epidural abscess to the right psoas muscle (arrows).

Outpatient MRI showed changes in L_1_-L_2_ consistent with infectious discitis, accompanied by a paravertebral collection extending onto the right psoas muscle (figure 5A). These findings were confirmed upon admission, when a new MRI was taken showing spondylodiscitis and L_1_-L_2_ discal abscess extending onto the right psoas muscle and anterior peridural space, with no compression of the dural sac (figure 5B).

Drainage and biopsy were performed, and the purulent discharge was sent for routine and mycobacteria culture. Given the negative results of conventional microbiological analyses, and the presence of compromised vertebral bodies, tuberculosis was suspected, no lung involvement was found. Both PCR and *M. tuberculosis* culture were positive, confirming vertebral and peridural tuberculosis.

She received antituberculosis treatment for six months, resulting in full recovery.

## Ethical considerations

The scientific publication of these cases was made under the ethical approval by the *Comité Corporativo de Ética en Investigación* at the *Hospital Universitario Fundación Santa Fe de Bogotá,* CCEI-10372-2019.

## Discussion

Even though Colombia is not part of the World Health Organisation's (WHO) top 30 high burden tuberculosis countries ^6^, it is one of 12 high priority countries out of 35 WHO member states in the Americas, because of its tuberculosis incidence rate ^6^. In 2021, there were 41 cases of tuberculosis per 100,000 people in Colombia, making this disease a serious public health problem for the country ^7^.

**Figure 6:**
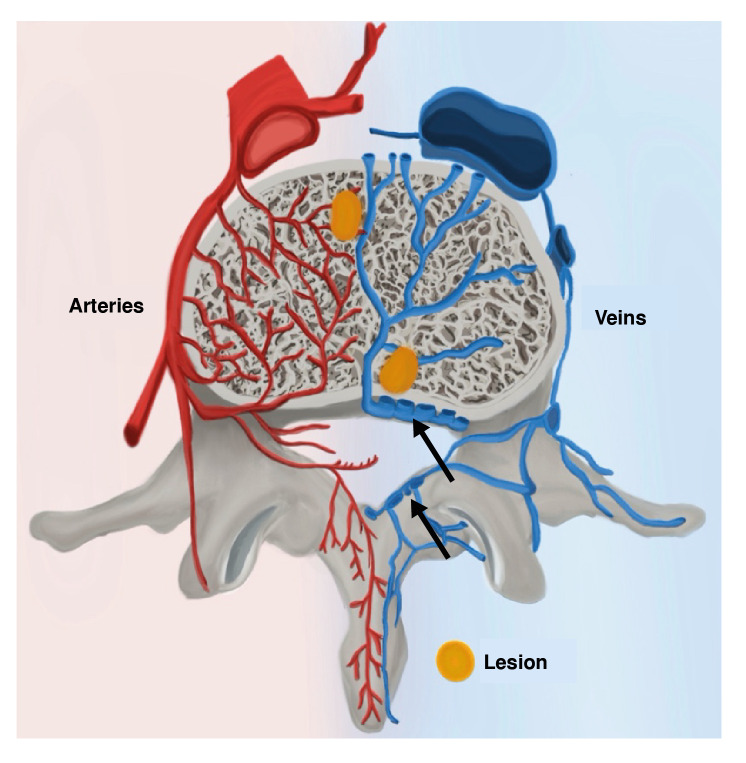
Diagram illustrating the routes of hematogenous dissemination of *Mycobacterium tuberculosis.* In red, a segmental artery branches off the aorta and gives rise to a central plexus. By this route, the mycobacteria access the anterior region of the vertebral body. Dissemination through Batson's venous plexus (arrows) leads to centrally originating lesions.

*Mycobacterium tuberculosis* may spread from the lungs to the spine despite the lack of radiologic and clinical evidence of pulmonary involvement, as was the case for all three patients presented ^4^. This process can occur via venous or arterial pathways ^4^. The arterial route originates from segmental branches of the aorta ^4^ (figure 6). These branches lead to anterior and posterior rami which form a subchondral plexus that facilitates access of the paradiscal space ^4^. Batson's paravertebral venous plexus is proposed as another avenue of dissemination ^4^ (figure 6). Without valves in these veins, blood flow depends on intra-abdominal and intrathoracic pressures ^4^. Hence, blood can drain either into intervertebral veins or deeper into the vertebral body ^4^.

When tuberculosis disseminates through the arteries, inflammation originates at the anterior, inferior, cancellous region of vertebral bodies ^3^^-^^5^^)^ (figure 6). Typically, multiple vertebrae are involved due to segmental arteries supplying more than one vertebra ^4^. Indeed, imaging studies conducted on our patients each showed involvement of adjacent vertebrae. If left untreated, the disease will spread and destroy the epiphyseal cortex, adjacent vertebrae, and even the intervertebral disk ^3^^-^^5^, causing spondylodiscitis, which can best be visualised in the first and third cases (figure 1 and 5). In many cases, infectious exudate and debris are released, accumulating in surrounding ligaments and muscles forming cold abscesses ^3^. The ensuing destruction of bone and cartilage leads to collapse of the vertebrae and, eventually, a form of structural kyphosis called gibbus deformity ^4^^-^^5^. The distorted bone structure can compress the spinal cord and cause neurological symptoms; a phase of the disease known as Pott's paraplegia ^5^.

Pott's paraplegia develops in almost a third of all patients with spinal tuberculosis ^3^. It is characterized by a gradual loss of function of the lower limbs, starting with weakness, numbness or paresis and progressing to full paralysis ^4^. Indeed, the first patient discussed here presented in the early stages of Pott's paraplegia.

Pott's paraplegia may occur during active disease or several years to decades later ^4^. Early onset paraplegia which occurs during active infection, is the result of several different pathological processes occurring simultaneously. These include direct compression of the spinal cord by vertebrae deformation, debris, pus and/or granulation tissue ^4^. The mid thoracic region is more susceptible to compression because the spinal canal is tighter around the cord ^5^. Moreover, destruction of the anterior portion of vertebrae causes subluxation and eventual dislocation, further damaging the spinal cord ^8^. Neurological deficits are also explained through *M. tuberculosis* infection of meninges and occlusion of blood vessels supplying the spinal cord ^4^. The physiopathology of late-onset paraplegia, also known as paraplegia of healed disease, is less understood but often associated with spinal deformities ^4^.

When the infection spreads through the venous system, osseous lesions may originate centrally and/or involve non-contiguous vertebrae ^4^ (figure 7). In general, these patients show no disk involvement ^4^. Vertebral body collapse leads to *vertebra plana,* or the complete compression of vertebral bodies ^4^^,^^9^. This is mostly seen in older-aged patients whose disks are less vascularised and less mineralized than younger-aged patients ^4^. This pathophysiological explanation corresponds with the presentation of our 61-year-old patient; her age and three separate lesions at T_9_-T_10_, L_4_-L_5_ and S_2_ point toward infectious spread through Batson's plexus (figure 2). The presentation seen in our first and third patients, with one lesion at T_10_-T_11_ and L_1_-L_2_, respectively, is suggestive of arterial dissemination (figures 1 and 5).

Pott's disease progresses slowly ^4^. Patients typically only consult upon experiencing severe pain, spinal deformity, or neurological symptoms ^4^. In cases two and three, however, patients sought medical treatment before the pain became very severe. Diagnoses were made at a later stage. The presentation of all three patients and common clinical findings are summarized in table 1.

The greatest challenge with respect to diagnosis is developing a clinical suspicion for tuberculosis ^5^. As was the case for all three patients presented, those with Pott's disease or articular tuberculosis often did not exhibit respiratory symptoms ^5^. Thus, knowledge of common skeletal tuberculosis manifestations is fundamental. History taking, including contact with previously diagnosed tuberculosis patients as well as immunosuppressive conditions, are important.

Plain radiographs and CT are helpful to evaluate the extent of spinal tuberculosis progression, anatomical distribution and, occasionally, to begin empirical treatment ^4^. X-ray findings are not specific ^4^, but in endemic countries, plain radiographs together with complete clinical evaluation are often enough to diagnose and empirically treat tuberculosis ^10^. However, X-rays only detect abnormalities at later stages ^4^. These findings include loss of end plate bone density, disk collapse, and osseous destruction. In some cases, paravertebral abscesses can be visualized ^4^. CT can show the osseous abnormalities and abscess formation before plain radiographs ^4^. MRI can show demineralisation of vertebral plates, loss of definition of bone margins, spinal deformities and abscesses ^4^. Early-stage MRI characteristics are less specific to tuberculosis but include disk degeneration and changes in bone marrow signal intensity ^4^. These findings are suggestive of bone marrow edema, which can be visualised in figures 1 and 2. As infection progresses, obliteration of disc space and vertebral collapse become apparent ^4^.

**Table 1:** Comparison of the described cases with the most common clinical presentations

	Patient one	Patient two	Patient three
	(66 years)	(61 years)	(37 years)
Systemic symptoms: fever, anorexia, weight loss, night sweats ^4^	None	None	None
Most common symptom: local back pain ^4^	Present	Present	Present
Other specific features: cold abscesses, gibbus (or other spinal deformity), muscle spasm ^4^	No spinal deformity, but a fracture	No spinal deformity	No spinal deformity
	No abscesses	No cold abscesses	Epidural cold abscess extending onto the right psoas muscle.
	No muscle spasms	No muscle spasms	
			No muscle spasm.
Two contiguous vertebrae involved. Lower thoracic and lumbar vertebrae involved ^4^	T_10_-T_11_	T_9_-T_10_, L_2_-L_3_, L_5_	L_1_-L_2_
Neurological deficits ^4^	Bilateral lower limb paresthesia	None	None

While there are no pathognomonic features of tuberculosis that appear on a CT scan, FDG PET-CT, a well-established diagnostic tool for cancer, has become an emerging technique in infectious diseases radiological diagnosis ^4^^-^^11^. FDG PET-CT captures the whole body ^11^ and can be used to distinguish between spinal tuberculosis and metastases, the latter often showing uptake at multiple levels ^4^. However, this distinction was not helpful in diagnosing patient two as tuberculosis caused multiple noncontiguous lesions. The maximum standardized uptake volume (SUV_max_) can be used to evaluate response to treatment and provide a quicker means of diagnosing antibiotic resistant tuberculosis ^11^. It is important to keep in mind that avascular regions may produce cold spots which would not produce the inflammatory response necessary for increased glucose uptake on a PET-CT.

The list of differential diagnoses for spinal tuberculosis is short. Two main diseases to keep in mind are spinal brucellosis and metastatic lesions ^5^^,^^12^^,^^13^. The main symptom of all these affections is back pain ^14^. Neurological deficits are most common in spinal tuberculosis ^14^. Plain radiographs are non-specific and unreliable in distinguishing between the illnesses. The different radiological findings of each of these three diagnoses are highlighted in table 2.

**Table 2:** Imaging findings of spinal tuberculosis, spinal brucellosis, and spinal metastases

	X-Rays	CT	MRI	PET-CT
Spinal tuberculosis	Vertebral body destruction, decreased disk height, sclerosis, hyperplasia ^4^	Early osteolytic destruction, sclerosis, paravertebral /epidural abscesses with calcifications ^4^	Changes in disco-vertebral signal intensity, spinal deformities, spinal cord compression ^4^	Possible accumulation of tracer usually at one level ^4^
Spinal brucellosis	Multiple vertebral body lesions, moth-eaten-like destruction, decreased disk height, hyperplasia, sclerosis ^13^	Early osteolytic destruction, bony bridges, sclerosis of endplates, and osteoporosis of vertebral bodies ^13^	Changes in disco-vertebral signal intensity, rare paravertebral/ epidural abscesses, spinal cord compression, Pedro Pons’ sign ^14^	--
Spinal metastases	Bone destruction, fracture, rare bone scalloping ^12^	Cortical destruction ^12^	Changes in vertebral signal intensity ^12^	Accumulation of tracer at multiple levels ^4^

CT: Computerized Tomography; MRI: Magnetic Resonance Imaging; PET-CT: Positron Emission Tomography-Computerized Tomography

Confirmational diagnosis of skeletal tuberculosis is made through histology or molecular analyses, or through culture of exudate or tissue ^4^^,^^5^. The currently used system for tuberculosis culture is mycobacteria growth indicator tube. Since this technique takes up to two weeks, the diagnosis of tuberculosis is in large part dependent on histological evidence, which confirms the diagnosis in -60% of patients ^4^. Epithelial, or necrotic granulomas are the hallmark histological findings ^4^. Along with radiological evidence, these characteristics are enough to begin treatment ^4^. polymerase chain reaction (PCR), while less accessible, can provide rapid diagnosis with better accuracy than histology ^4^.

Skeletal tuberculosis is entirely curable with better prognosis when it is diagnosed and treated promptly ^4^. Treatment involves antibiotics and surgical intervention for cases with advanced vertebral lesions ^4^^,^^5^. Tuberculosis' status as a major public health issue ^3^ suggests that management is not as simple as it sounds. Indeed, tuberculosis has long been described as a social disease, making its appearance in times of socio-economic instability. Scientifically advanced diagnostic and treatment techniques are not accessible to everyone or, in most cases, to those who need them the most ^3^^,^^5^. Efforts to treat this illness have also faced the increasing challenge of antibiotic resistance ^3^^,^^5^.

We hope the discussion of these case studies helps increase awareness of the signs, symptoms, and radiological findings of skeletal tuberculosis to facilitate early diagnosis and prompt treatment.

## Conclusion

The three cases highlight the variety of presentation and diagnostic challenges of spinal tuberculosis. Pott's disease is the result of arterial and/or venous dissemination of *M. tuberculosis* to the spine. Each of these avenues lead to vertebral destruction and spread of infection to surrounding structures. Patients most commonly present with back pain. Pott's disease requires a high index of suspicion as the clinical presentation can mimic other spinal pathologies, such as spinal metastases.

In patients older than 60 years old who present with back pain and have spinal MRI imaging that show affectation of more than one vertebra, adjacent or not, with disc involvement, skeletal tuberculosis must be ruled out. Without treatment, patients will experience neurological symptoms known as Pott's paraplegia, which begin in the lower limbs and slowly progress to full paralysis.

Early diagnosis of spinal tuberculosis is crucial to prevent these complications. Imaging studies, such as MRI, play a pivotal role in detecting characteristic findings of Pott's disease. Microbiological confirmation is essential for definitive diagnosis.

## References

[1] World Health Organization (2024). Global tuberculosis report 2024.

[2] Center for Disease Control and Prevention Clinical overview of tuberculosis disease.

[3] Sharma SK, Mohan A, Kohli M (2021). Extrapulmonary tuberculosis. Expert Rev Respir Med.

[4] Garg RK, Somvanshi DS (2011). Spinal tuberculosis: A review. J Spinal Cord Med.

[5] Stout J (2022). Bone and joint tuberculosis. UpToDate.

[6] World Health Organization (2021). Global lists of high burden countries for tuberculosis (TB), TB/HIV and multidrug/rifampicin-resistant TB (MDR/RR-TB), 2021-2025: Background document.

[7] World Bank (2024). Incidence of tuberculosis.

[8] Sternbach G. (1996). Percivall Pott: Tuberculous spondylitis. J Emerg Med.

[9] Glassman I, Nguyen KH, Giess J, Alcantara C, Booth M, Venketaraman V (2023). Pathogenesis, diagnostic challenges, and risk factors of Pott’s disease. Clin Pract.

[10] Semionov A, Lebel K, Diouf A, Pressacco J (2022). Tuberculosis: A head-to-toe radiological review. Open J Radiol.

[11] Manika K, Kipourou M, Georga S, Faniadou E, Pilianidis G, Arsos G (2020). 18F-FDG PET/ CT contribution to tuberculous vertebral osteomyelitis diagnosis: a case report. Oxf Med Case Rep.

[12] Shah LM, Salzman KL (2011). Imaging of spinal metastatic disease. Int J Surg Oncol.

[13] Tu L, Liu X, Gu W, Wang Z, Liu Z, Zhang E (2018). Imaging-assisted diagnosis and characteristics of suspected spinal brucellosis: A retrospective study of 72 cases. Med Sci Monit.

[14] Rizkalla JM, Alhreish K, Syed IY (2021). Spinal brucellosis: A case report and review of the literature. J Orthop Case Rep.

